# Retraction Note: Osteocyte-derived exosomes induced by mechanical strain promote human periodontal ligament stem cell proliferation and osteogenic differentiation via the miR-181b-5p/PTEN/AKT signaling pathway

**DOI:** 10.1186/s13287-024-04031-5

**Published:** 2024-11-05

**Authors:** Pei-ying Lv, Peng-fei Gao, Guang-jie Tian, Yan-yan Yang, Fei-fei Mo, Zi-hui Wang, Lu Sun, Ming-jie Kuang, Yong-lan Wang

**Affiliations:** 1 Department of Periodontology, School and Hospital of Stomatology, Tianjin Medical University, Tianjin, 300070 China; 2 Department of Periodontology, The Affiliated Stomatology Hospital of Soochow University, Suzhou, 215000 Jiangsu China; 3 Department of Oral Medicine, Infection and Immunity, Harvard University School of Dental Medicine, Boston, MA 02115 USA; 4 Department of Orthopedics, The Provincial Hospital Affiliated to Shandong First Medical University, Jinan, 250014 Shandong China

**Retraction Note: Stem Cell Research & Therapy (2020) 11:295**

10.1186/s13287-020-01815-3

The Editor-in-Chief has retracted this article. The authors of this article subsequently contacted the journal to request to replace components of Figs. 1B, 6E, 6H and 7A. Further investigation raised concerns regarding the provenance of the replacement figures and the rationale behind the need to replace these figures. The Editor-in-Chief therefore no longer has confidence in the reliability of the data reported in this article.

Author Yong-lan Wang has stated that all authors disagree with this retraction.

